# Suppressing the PI3K/AKT Pathway by miR-30d-5p Mimic Sensitizes Ovarian Cancer Cells to Cell Death Induced by High-Dose Estrogen

**DOI:** 10.3390/biomedicines10092060

**Published:** 2022-08-24

**Authors:** Alexandra Varga, Éva Márton, Arnold Markovics, András Penyige, István Balogh, Bálint Nagy, Melinda Szilágyi

**Affiliations:** 1 Department of Human Genetics, Faculty of Medicine, University of Debrecen, H-4032 Debrecen, Hungary; 2 Institute of Food Technology, Faculty of Agricultural and Food Sciences and Environmental Management, University of Debrecen, H-4032 Debrecen, Hungary; 3 Faculty of Pharmacy, University of Debrecen, H-4032 Debrecen, Hungary; 4 Division of Clinical Genetics, Department of Laboratory Medicine, Faculty of Medicine, University of Debrecen, H-4032 Debrecen, Hungary

**Keywords:** miR-30, ovarian cancer, estrogen receptor, high-dose estrogen, cell proliferation, cell death, SOX4, PI3K/AKT, cancer therapy, tamoxifen

## Abstract

MicroRNAs are short non-coding RNA molecules that are involved in tumor development and are considered to be promising candidates in cancer therapy. Here, we studied the role of miR-30s in the pathophysiology of ovarian cancer. According to our results miR-30a-5p, miR-30d-5p, and miR-30e-5p were overexpressed in the estrogen receptor α (ERα)-expressing PEO1 cell line compared to A2780 that lacks this receptor. Furthermore, the expression of miR-30a-5p, miR-30d-5p, and miR-30e-5p were induced in response to high-dose estrogen treatment in PEO1 where intensive cell death was observed according to the induction of apoptosis and autophagy. Lacking or blocking ERα function reduced tolerance to high-dose estrogen that suggests the importance of ERα-mediated estrogen response in the maintenance of proliferation. MiR-30d-5p mimic reduced cell proliferation in both A2780 and PEO1. Furthermore, it decreased the tolerance of PEO1 cells to high-dose estrogen by blocking the ERα-mediated estrogen response. This was accompanied by decreased *SOX4* expression that is thought to be involved in the regulation of the PI3K/AKT pathway. Blocking this pathway by AZD8835 led to the same results. MiR-30d-5p or AZD8835 sensitized PEO1 cells to tamoxifen. We suggest that miR-30d-5p might be a promising candidate in the therapy of ovarian cancer.

## 1. Introduction

According to the latest statistics, ovarian cancer is the eighth most common type of cancer among females with more than 313,000 new cases and 207,000 deaths annually worldwide (3.4% and 4.7% of all cancer types, respectively) [1]. The 5-year survival rate has significantly increased recently in several cancer types due to advances in screening and treatment options [2]. However, the survival rate in ovarian cancer has shown a minor increase only due to the fact that 75% of cases are still diagnosed in an advanced stage where the survival rate is only 20% [3]. The acquired chemoresistance against the applied chemotherapeutic agents—that occurs frequently in advanced stage cancer—represents another reason for the high mortality rate of ovarian cancer [4,5]. Although several modern therapeutics are available or are under investigation for the better treatment of ovarian cancer (e.g., including angiogenesis and poly (ADP-ribose) polymerase inhibitors or immunotherapy agents), platinum-based chemotherapy is the first-line therapeutic agent [3,4,6]. Endocrine therapy, including the use of estrogen blockers/antagonists (e.g., tamoxifen) or aromatase inhibitors (e.g., letrozole), represents another cost-effective therapeutic option in estrogen-sensitive tumors that has significantly improved survival rates in breast cancer [7].

MicroRNAs (miRNAs) can be defined as ~18–24 bp long non-coding RNAs that act in the post-transcriptional regulation of gene expression [8]. These molecules are confirmed to be important regulators of cancer development and progression due to the fact that they might function either as tumor suppressors or oncogenes [9,10]. They are considered to be promising biomarker candidates in cancer diagnostics that is based on the observation that the miRNA expression pattern of tumor cells differs from healthy cells. Determining cell-free miRNA expression in body fluids makes miRNAs suitable candidates for non-invasive diagnostics as well that supports screening and follow-up of the disease [11,12]. Furthermore, modulating miRNA expression in tumor cells considered to be a promising therapeutic strategy in cancer. Replenishing tumor suppressor miRNAs by miRNA mimics or suppressing oncogenic miRNAs by the application of antisense oligonucleotides or antagomirs proved to be effective in suppressing tumor growth and invasion potential [13,14]. Several clinical studies about miRNA-based therapeutic options are in progress [15]. However, their application in everyday clinics is still hindered due to the following challenges: (i) it is difficult to identify the best candidate miRNAs, (ii) it requires the development of vehicles for the efficient delivery of miRNA therapeutics to target cells, and (iii) it is necessary to find the optimal doses/combinations to avoid toxicity and off-target effects [14,16]. Due to the rapidly growing interest that has been invested to find applicable candidates, characterizing miRNAs has become a hot topic in cancer research recently. Furthermore, several transfection methods have been developed for the efficient delivery of miRNAs including viral (e.g., by adeno-associated virus) and non-viral systems (e.g., by lipid, polymeric, or bio-conjugated nanocarriers). The chemical modification (e.g., adding methyl or phosphorothioate-like groups) of miRNAs is also available that increases their stability and reduces their toxicity [14,16]. Hence, the clinical application of miRNA-based therapeutics might not be too far away.

Previously, we have identified several miRNAs that might have high biological relevance in estrogen-sensitive ovarian cancer cells and are suggested to be promising non-invasive biomarkers in ovarian cancer [17,18,19,20]. Here, our aim was to characterize the biological relevance of miR-30 family members in ovarian cancer cells. These are considered to be important tumor suppressors in several cancers [21]. However, their role in ovarian cancer is understudied. Our aim was to characterize these miRNAs in ovarian cell cultures. We have found that miR-30s showed higher basal expression in the PEO1 estrogen receptor α (ERα)-expressing ovarian cell line and the expression of miR-30a-5p, miR-30d-5p, and miR-30e-5p was markedly elevated in response to high-dose estrogen treatment where intensive cell death was observed. The application of miR-30d-5p mimic reduced cell proliferation in PEO1 and A2780 cells. Furthermore, miR-30d-5p mimic sensitized PEO1 cells to cell death that is induced by high-dose estrogen, which was in good agreement with reduced *SOX4* expression that is suggested to be involved in the regulation of the PI3K/AKT pathway. The application of miR-30d-5p sensitized the PEO1 cells to tamoxifen. We conclude that miR-30d-5p alone, or in combination with other agents (e.g., with tamoxifen) might be a promising candidate in the therapy of ovarian cancer.

## 2. Materials and Methods

### 2.1. Culturing Conditions

We applied the following human epithelial ovarian cell cultures in this study: PEO1 that was purchased from Merck (Darmstadt, Germany; ECACC, Salisbury, UK) and highly expressed ERα according to previous studies [18,22]. The other cell line was A2780, which was kindly provided by Katalin Goda (Department of Biophysics and Cell Biology, Faculty of Medicine, University of Debrecen, Debrecen, Hungary) in which ERα expression was not observed previously [18,22]. The cell lines were routinely cultured in RPMI1640 (Corning, New York, NY, USA) that was supplemented with 10% Fetal Bovine Serum (FBS, Corning, New York, NY, USA), 1% L-glutamine (Corning, New York, NY, USA), 100 µg/mL streptomycin (Corning, New York, NY, USA), and 100 U/mL penicillin (Corning, New York, NY, USA; 37 °C, 90% humidity, 5% CO_2_). When the effect of estrogenic compounds was tested, exponentially growing cultures were plated to 96- or 24-well plates after harvesting by trypsinization and cultured overnight in RPMI1640 that was supplemented with 10% FBS. Note that in order to reduce the confounding effect of the different doubling times of the applied cell lines the plates were inoculated with different cell numbers: A total of 5000 or 50,000 cells were applied in the case of A2780 and 10,000 or 100,000 cells were used in the case of PEO1 in the 96- or 24-well plates, respectively. Then medium was replaced with PRF-RPMI1640 (Corning, New York, NY, USA) that was supplemented with 5% DCC-FBS (Corning, New York, NY, USA) in order to decrease the confounding effect of estrogens that were present in the conventional culturing medium. After 24 h of incubation the cultured cells were treated with estradiol (E2) (Merck, Darmstadt, Germany) in 10 nM, 1, 10, 20, 30, 40, 50, and 100 µM final concentrations and that time point was designated as 0 h. In some experiments, the cells were also treated with methyl-piperidino-pyrazole (MPP; Merck, Darmstadt, Germany; 10 nM), AZD8835 (100 nM), tamoxifen (Merck, Darmstadt, Germany; 1 µM) or 4-[2-Phenyl-5,7-bis(trifluoromethyl)pyrazolo [1,5-a]pyrimidin-3-yl]phenol (PHTPP; Merck, Darmstadt, Germany; 10, 100 nM, 1, 10 µM).

### 2.2. Determination of Cell Proliferation

Cell proliferation was tested by MTT assay. The cells were plated to 96-well plates and treated with estrogens as previously described. During cell proliferation testing, 90 µL fresh medium that was supplemented with 10 µL 3-(4,5-Dimethylthiazol-2-yl)-2,5-Diphenyltetrazolium Bromide (MTT; Merck, Darmstadt, Germany; 5 mg/mL in PBS) was added to the wells. After incubation (4 h, 37 °C), the MTT solution was removed and formazan crystals were dissolved with 100 µL solubilization solution (81 *v*/*v*% 2-propanol, 9 *v*/*v*% 1 M HCl, 10 *v*/*v*% Triton-X 100; Merck, Darmstadt, Germany). A Multiskan sky microplate reader (Thermo Fisher Scientific, Walthman, MA, USA) was applied to determine the absorbance at 567 nm. The cell proliferation in response to the treatments was designated relative to the non-treated control (1) from 4 independent experiments.

### 2.3. Determination of Mitochondrial Membrane Potential

The occurrence of apoptosis was determined by a DilC1(5) assay that is widely used to monitor mitochondrial membrane potential whose decrease is an early marker for apoptosis, as described elsewhere [23]. A CLARIOstar Plus microplate reader (BMG Labtech, Ortenberg, Germany) was used to measure the fluorescence signal at 630 nm excitation and 670 nm emission. The rate of apoptosis in response to the treatments was designated relative to the non-treated control (1) that was determined from 4 independent experiments.

### 2.4. Determination of Cell Lysis

The presence of cell lysis was quantified by detecting the activity of lactate dehydrogenase (LDH) enzyme in the fermentation broth of cell cultures using the CyQUANT LDH Cytotoxicity Assay Kit (Thermo Fisher Scientific, Walthman, MA, USA) following the protocol that was provided by the manufacturer. LDH is supposed to be an intracellular enzyme, thus the increase of LDH activity in the fermentation broth of the cultures suggests cell lysis. Enzyme activity was determined at 490 nm using the Multiskan sky microplate reader (Thermo Fisher Scientific, Walthman, MA, USA). Cell lysis in response to the treatments was determined relative to the non-treated control (1) from 4 independent experiments.

### 2.5. MRNA Isolation and Quantification

In gene expression studies, the cells were cultured in 24-well plates and treated with E2 in different concentrations as previously described. A total of 24 h after the treatment, the total RNA was isolated with the Quick-RNA MiniPrep Kit (Zymo Research, Irvine, CA, USA) according to the manufacturer’s instructions. A total of 500 ng total RNA was applied for cDNA synthesis using the Maxima First Strand cDNA Synthesis Kit (Thermo Fisher Scientific, Walthman, MA, USA) following the steps: 25 °C 10 min, 50 °C 15 min, and 85 °C 5 min. The total RNA and cDNA concentrations were determined by NanoDrop LITE Spectrophotometer (Thermo Fisher Scientific, Walthman, MA, USA). The expression of *GREB1*, *CA12*, *TP53*, *ATG2B*, *ATG12*, *BAG3*, *SOX4,* and *ESR2* was quantified by qPCR by the Maxima™ SYBR Green qPCR Master Mix (Thermo Fisher Scientific, Walthman, MA, USA) according to the manufacturer’s instructions using 200 ng cDNA. The PCR program included the following steps: denaturation at 95 °C for 10 min, followed by 40 cycles of 94 °C for 15 s, 60 °C for 30 s, and 72 °C for 30 s. Finally, a melting curve was generated by taking fluorescent measurements from 65 °C until 97 °C to ensure the detection of a single PCR product. The quantification was performed in triplicates. *GAPDH* was used to normalize the expression values using the results of 4 independent experiments. In order to characterize the changes in gene expression in response to E2 treatment, relative expression ratios were determined relative to the non-treated control samples using the 2^−ΔΔCt^ formula. The FC values are presented in log_2_ values. The primer sequences are listed in Supplementary Table S1.

### 2.6. MiRNA Isolation and Quantification

In these studies, cells were plated to 24-well plates and exposed to E2 as previously described. A total of 24 h after the treatment we isolated total RNA including small RNAs by the miRNeasy Kit (Qiagen, Hilden, Germany) according to the protocol that was provided by the manufacturer. Determination of the miRNA concentration was performed by miRNA-specific fluorometric assay using a Qubit^®^ 2.0 Fluorometer (Thermo Fischer Scientific, Waltham, MA, USA). In the expression analysis of miR-30a-3p, miR-30a-5p, miR-30d-5p, and miR-30e-5p the miScript workflow was applied. A total of 10 ng miRNA was used for reverse transcription by the miScript II RT Kit (Qiagen, Hilden, Germany), then the expression of the specified miRNAs was determined by the miScript SYBR Green PCR Kit (Qiagen, Hilden, Germany) with miRNA specific primer assays (miScript primer assays; Qiagen, Hilden, Germany) following the protocol that was provided by the manufacturer. The Lightcycler 96 instrument (Roche, Pleasanton, CA, USA) was applied for the analysis. A total of 500 pg cDNA was added to the reaction mixture. The steps of the PCR were: denaturation at 95 °C (15 min) followed by 50 cycles of 94 °C (15 s), 55 °C (30 s), and 70 °C (30 s). In the end, melting curve analysis was performed in order to ensure the detection of a single PCR product by measuring the fluorescence between 40 °C and 85 °C. The quantification was performed in triplicates. The expression of miR-103-3p was used as an internal reference that was also applied in our previous experiments [17,18,19,20]. The normalized expression was determined using the results of 4 independent experiments applying the 2^−ΔCt^ formula.

### 2.7. Transfection of PEO1 Cultures with miRNA Mimic

In order to characterize miR-30d-5p function, miRIDIAN microRNA Mimic (Horizon, Cambridge, UK) was applied. PEO1 and A2780 cells were plated to 96- or 24-well plates as described earlier. After the attaching medium was removed to PRF-RPMI1640 (Corning, New York, NY, USA) that was supplemented with 5% DCC-FBS (Corning, New York, NY, USA) and cells were transfected with miR-30d-5p mimic or miRIDIAN microRNA Mimic Negative Control (Horizon, Cambridge, UK) in various doses (25, 50, 100 nM) using DharmaFECT 1 siRNA Transfection Reagent (Horizon, Cambridge, UK) following the manufacturer’s instructions. A total of 24 h later the medium was changed to fresh medium and transfection was repeated. This time point was designated as 0 h. In some experiments, PEO1 cultures were also treated at this time point with E2 (10 nM, 1–100 µM), AZD8835 (100 nM), or tamoxifen (1 µM).

### 2.8. Statistical Analysis

Statistical significance of the treated and non-treated cell cultures was determined by a Student’s t-test or one-way ANOVA with Dunnet’s test as post hoc analysis. Statistical analysis and figures were made by GraphPad Prism 7.

### 2.9. Functional Annotation and Pathway Enrichment of miR30s and Their Targets

At first, the common, validated target genes of the four dysregulated miRNAs were assembled from the miRTarBase 8.0 (http://miRTarBase.cuhk.edu.cn, accessed on 1 November 2021) database, using the miRNA Enrichment Analysis and Annotation Tool (miEAA 2.0; https://ccb-compute2.cs.uni-saarland.de/mieaa2/, accessed on 1 November 2021). The web-based miRPathDB 2.0 tool (https://mpd.bioinf.uni-sb.de, accessed on 1 November 2021) was used to collect the experimentally validated target genes of the investigated miRNAs by applying the miRTarBase 8.0 (http://miRTarBase.cuhk.edu.cn, accessed on 1 November 2021) database in order to carry out an enrichment and functional annotation of the specified miRNA targets in biological pathways by the miRPathDB 2.0 tool. A heatmap was generated to visualize all the functional categories that are significantly enriched for the targets of at least one of the miRNAs. To identify the enrichment of miRNA targets in biological pathways, the tool uses the GeneTrail2 C++ library and we searched for pathways in the Reactome database. The miRNA-target gene and general protein–protein interaction network of target genes was also constructed using the miRNet tool (http://www.mirnet.ca, accessed on 5 November 2021). To construct the network, the experimentally validated target genes that were specified by the miRNet tool were used. MiRNet integrates data from TarBase, miRTarBase, miRecords, miRanda, miR2Disease, HMDD, PhenomiR, SM2miR, PharmacomiR, EpimiR, and starBase databases. The protein–protein interaction network of target genes that were included in the network was constructed using the STRING database (http://string-db.org, accessed on 5 November 2021). The top miRNAs and the target genes in the network were ranked by degree and betweenness centrality values.

## 3. Results

### 3.1. MiR-30 Family Members Are Overexpressed in the ERα Expressing PEO1 Ovarian Cells and Their Expression Responded to Estrogen Treatment

**Figure 1:**
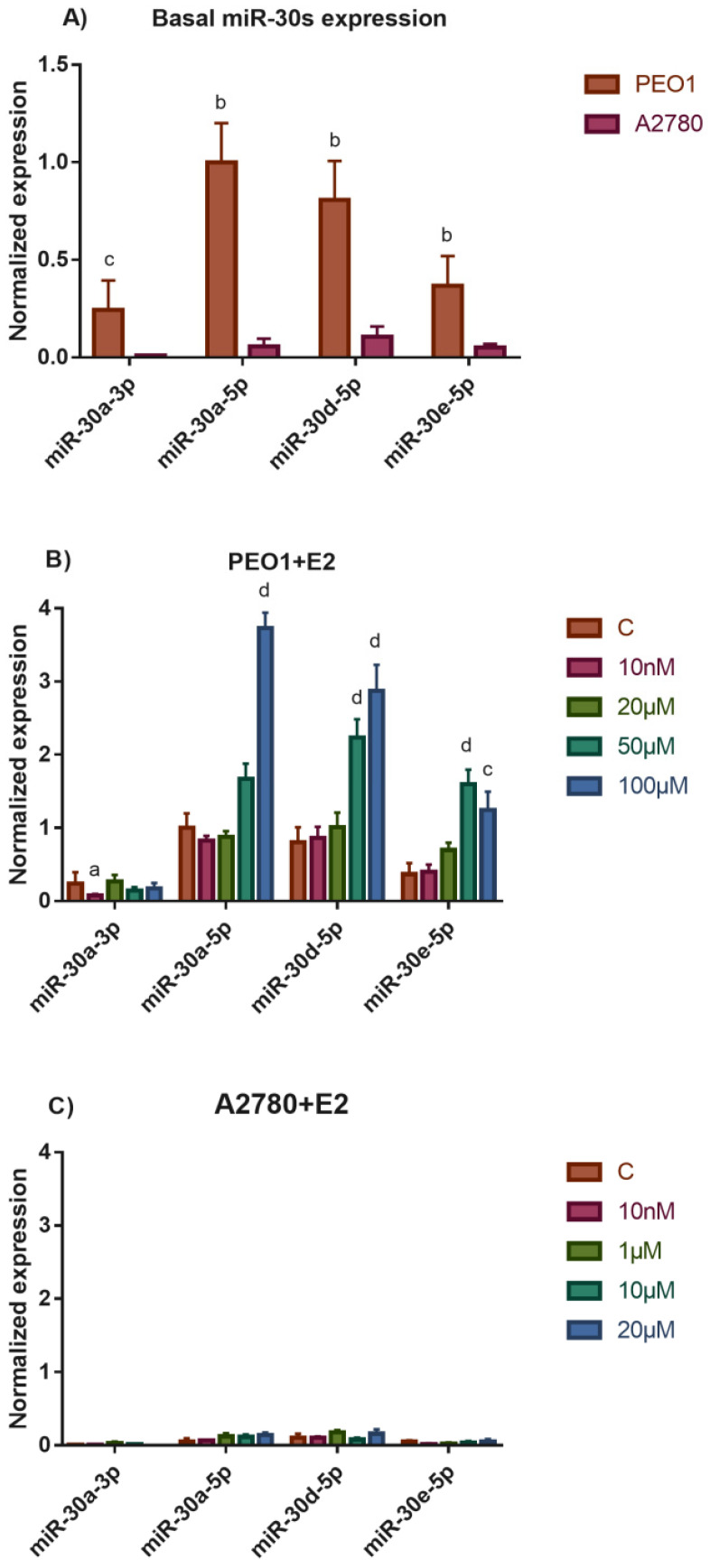
Study the expression of miR-30s in the PEO1 and A2780 cell lines. (**A**) Comparison of basal miR-30s expression in the PEO1 and A2780 cell lines. (**B**) Normalized expression of miR-30s in the PEO1 cell line in response to E2 treatment. (**C**) Normalized expression of miR-30s in the A2780 cell line in response to E2 treatment. The data are presented as the mean ± S.D. The ΔCt values were used to calculate statistical significance. a: *p* < 0.05; b: *p* < 0.01; c: *p* < 0.001; d: *p* < 0.0001.

There were two human epithelial ovarian cell lines that were used in our study. The ERα-positive PEO1 cell line and the A2780 cell line that does not express ERα [18]. According to our previous studies, ERα expression strongly influenced the basal expression of miRNAs in these cell lines [18,19]. Based on the above findings, we further investigated the basal expression of miR-30a-3p, miR-30a-5p, miR-30d-5p, and miR-30e-5p in the PEO1 and A2780 cell lines. MiR-30s showed significantly higher basal expression in PEO1 than in A2780 cells (Figure 1A). Among these, miR-30a-5p and miR-30d-5p had the highest basal expression level in PEO1 (Figure 1A). These results suggest that miR-30s—especially miR-30a-5p and miR-30d-5p—might have higher biological relevance in the ERα-expressing PEO1 cell line. We also determined whether the expression of miR-30s change in response to estrogen exposure, a phenomenon that was observed in the case of some miRNAs in our previous studies [18,19]. In these experiments, the PEO1 cell line was treated with E2 in a 10 nM–100 µM concentration range. Only miR-30a-3p responded to the physiologically relevant 10 nM E2 dose, where the expression was significantly decreased. In contrast to miR-30a-3p, the expression of miR-30a-5p, miR-30d-5p, and miR-30e-5p was not affected by low-dose E2 treatment. According to this observation, these miRNAs might not be involved in the ERα-mediated induction of cell proliferation in response to 10 nM E2. However, the expression of these miRNAs markedly increased in response to higher (50 and 100 µM) E2 doses in the PEO1 cells (Figure 1B); these doses were able to induce cell death previously [24]. This supposes that miR-30a-5p, miR-30d-5p, and miR-30e-5p might have a role in the regulation of cell death instead in PEO1. Note that the relatively low expression of miR-30s did not change significantly in response to E2 treatment in the A2780 cells (Figure 1C).

### 3.2. High-Dose E2 Treatment Inhibits Cell Proliferation and Induces Cell Death That Is Highly Influenced by the Induction of ERα-Mediated Estrogen Response

In order to shed light on the biological significance of miR-30s in response to high-dose E2 in PEO1 cells, we characterized the phenotypic effect of high-dose estrogen to ovarian cells. In these experiments, the A2780 cell line was applied as an ERα non-expressing negative control. The response of cell cultures to E2 treatment was determined by assessing the following parameters: (i) the extent of cell proliferation, (ii) the decrease of mitochondrial membrane potential that is considered to be an early marker of apoptosis, and (iii) the level of LDH activity in the supernatant of cell cultures indicating cellular lysis. Furthermore, gene expression changes were also determined in response to the treatments. To characterize ERα-mediated estrogen response, the expression of *GREB1* and *CA12*—shown to be useful markers of estrogen response in our previous studies [18]—was tested. In order to characterize whether cell death was mediated via apoptosis or autophagy, the expression of *TP53* was studied as a marker for apoptosis as well as that of *ATG2B*, *ATG12*, and *BAG3* that are involved in autophagy. All of these genes are considered to be under the regulation of ERs [25,26,27]. The expression of *SOX4,* a confirmed target of miR-30d-5p, was also determined [28].

**Figure 2:**
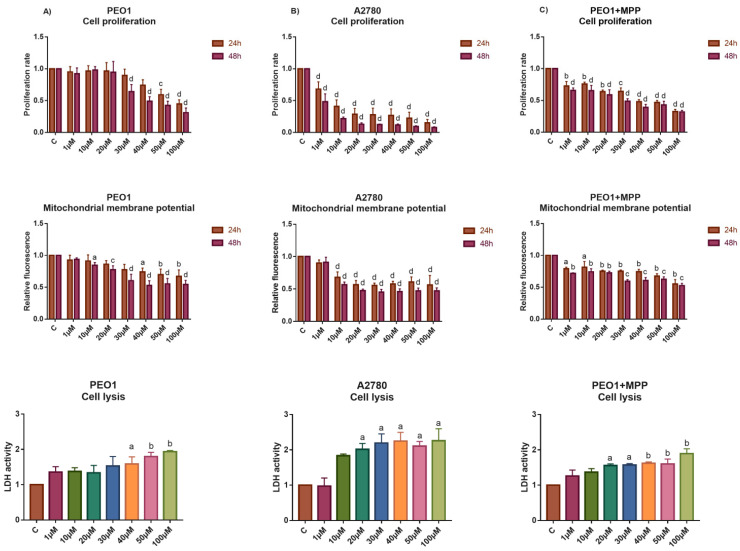
Study the effect of high-dose E2 exposure to the cell proliferation, apoptosis and cell lysis of the PEO1 and A2780 cell cultures. Phenotypic characterization of high-dose E2 treatment was made in the PEO1 (**A**) and A2780 (**B**) cell lines as well as in the PEO1 cell line in the presence of 10 nM MPP (**C**). The rate of cell proliferation, mitochondrial membrane potential (24 h and 48 h after the treatment), and LDH activities (48 h after the treatment) were determined relative to the non-treated control (1). The data are presented as the mean ± S.D. a: *p* < 0.05; b: *p* < 0.01; c: *p* < 0.001; d: *p* < 0.0001.

**Figure 3:**
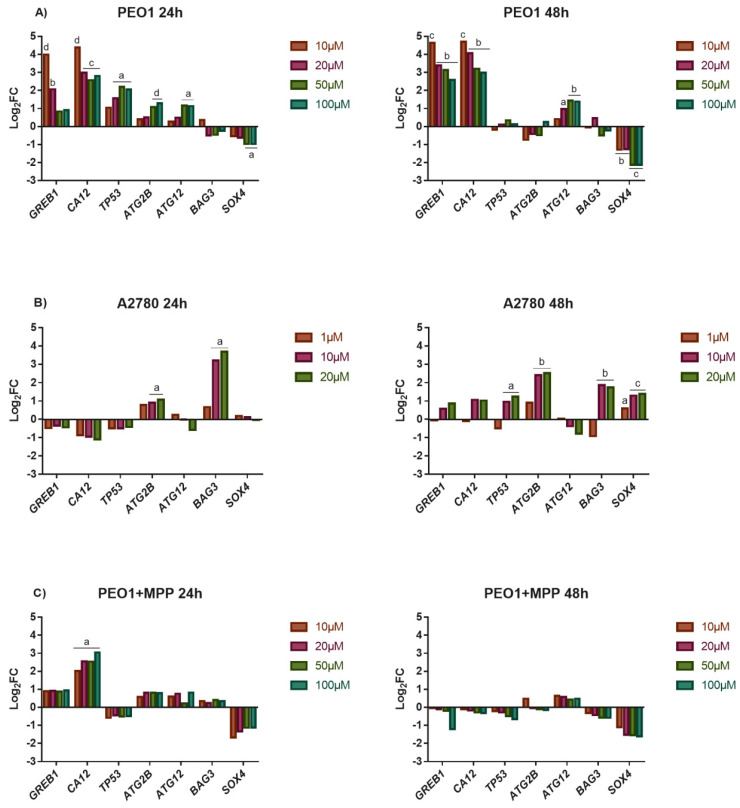
Study the effect of high-dose E2 exposure on gene expression. Log_2_FC values of the selected genes were determined in response to E2 treatment in the PEO1 (**A**) and A2780 (**B**) cell lines as well as in the PEO1 cell line in the presence of 10 nM MPP (**C**). The total RNA was isolated from the cells at 24 h and 48 h after E2 treatment. Significance was determined between the ∆CT values of the treated and non-treated samples. a: *p* < 0.05; b: *p* < 0.01; c: *p* < 0.001; d: *p* < 0.0001.

As Figure 2A presents, the PEO1 cells tolerated the lower 10–20 µM doses of E2 well, where significant changes in miR-30s expression were not observed. The elevated expression of *GREB1* and *CA12* genes suggests the involvement of ERα-mediated response even in these E2 doses (Figure 3A). However, cell proliferation was inhibited and cell death was induced at higher E2 doses (Figure 2A). According to the lower response of *GREB1* and *CA12* to 50 and 100 µM doses of E2, the ERα-mediated response proved to be less relevant in these doses. The detected depolarization of mitochondrial membrane potential suggests the role of apoptosis in PEO1 cells following E2 treatment that was also suggested by the dose-dependent induction of the *TP53* gene (Figure 2A and Figure 3A). The induction of *ATG12* and *ATG2B* genes supposes that autophagy was also involved in the cell death of PEO1 (Figure 3A). It is also important to mention that the dose-dependent repression of *SOX4* correlated well with the increased miR-30d-5p expression at 50 and 100 µM E2 doses (Figure 1B and Figure 3A).

Unexpectedly, the ERα non-expressing A2780 cell line proved to be more sensitive to the toxic effect of high-dose estrogens. Cell proliferation was reduced even at 1 µM dose and 48 h after the treatment intensive cell lysis was observed (Figure 2B). According to our gene expression analysis, the induction of *GREB1* and *CA12* was not experienced, which is in good agreement with our previous studies (Figure 3B) [18]. On the other hand, the significant induction of *ATG2B* and *BAG3* expression indicates that the intensive cell death process of A2780 cells might be attributed mostly to autophagy in response to the high-dose E2 treatment (Figure 3B).

We hypothesized that the higher sensitivity of A2780 to cell death that was induced by high-dose estrogens might be explained by the lack of ERα-mediated response in this cell line. In order to prove this hypothesis, high-dose E2 treatment was also performed in PEO1 in the presence of 10 nM MPP, which is a highly selective ERα antagonist [29]. The addition of MPP abolished the inductive effect of E2 to cell proliferation, which is in good agreement with the significantly lower expression of *GREB1* and *CA12* in response to E2 treatment (Figure 3C). As a consequence, the tolerance of PEO1 cells was markedly decreased to high-dose E2 treatment (Figure 2C and Figure 3C). Note that the application of 10 nM MPP solely did not change the expression of miR-30s or the genes that were tested (Supplementary Table S2).

We also considered whether the intensive cell death of A2780 in response to E2 was mediated by ERβ, which is considered to be a tumor suppressor [30]. In this case, the induction of ERβ by E2 might have resulted in the block of cell proliferation. A2780 had a low but detectable ERβ expression: 0.001613 ± 0.001 (relative to *GAPDH*). In these experiments we applied PHTPP, which is an ERβ-specific antagonist [31]. According to our results, blocking ERβ did not have any significant effect to the cell death mechanism of A2780 in response to high-dose E2 exposure (Supplementary Figure S1).

### 3.3. Bioinformatic Analysis of miR-30a-3p, miR-30a-5p, miR-30d-5p, and miR-30e-5p

**Figure 4:**
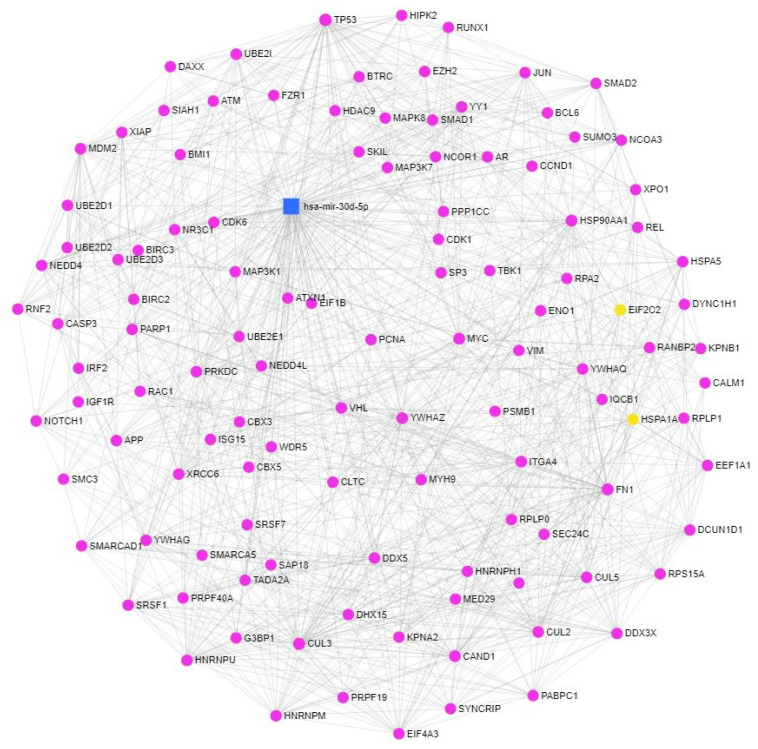
Functional annotation analysis of the targets of miR-30d-5p. Gene–gene and protein–protein interactions of the targets of miR-30d-5p were generated by the miRNet tool.

The induction of miR-30a-5p, miR-30d-5p, and miR-30e-5p in response to high-dose estrogen treatment, where intensive cell death was observed, suggests that these miRNAs might be involved in the regulation of cell proliferation and/or cell death. In order to prove this hypothesis, bioinformatic analysis was performed. Due to the fact that one miRNA might target several mRNAs and one mRNA might be targeted by several miRNAs, the regulation of gene expression is very likely mediated by miRNA networks rather than individual miRNAs [32]. As a consequence, the functional interaction between miRNAs might be defined by the number of their common targets. Functional analysis of the shared targets of miR-30a-3p, miR-30a-5p, miR-30d-5p, and miR-30e-5p was carried out by the miRNA Enrichment and Annotation (miEAA 2.0) webtool using the miRTarBase v8.0 database. According to our analysis, miR-30a-5p, miR-30d-5p, and miR-30e-5p share several targets, however, only a few of these targets are shared with miR-30a-3p (Supplementary Figure S2). This observation is in line with the distinct expression pattern of miR-30a-3p during our experiments. Functional enrichment analysis was also carried out in order to identify the molecular pathways where the targets of miR-30s are significantly enriched using the miRPathDB tool using the miRTarBase v8.0 database. Only a few hits were found for the overlapping miR-30a-3p and miR-30a-5p targets (Supplementary Figure S3). However, several pathways that were involved in cancer development were identified when the common targets of miR-30d-5p and miR-30e-5p were analyzed. In these pathways, the targets of miR-30d-5p were highly enriched in several processes that were involved in the regulation of cell death (apoptosis, autophagy) and senescence (Supplementary Figure S3). Therefore, miR-30d-5p was chosen for further network analysis. A network that was based on miRNA-target genes and protein–protein interactions was constructed with the miRNet tool using the targets of miR-30d-5p where the degree and betweenness centrality values were used to characterize the significance of the identified genes in the network (Table 1, Figure 4). According to these values, *TP53* might have the highest significance in the network. Functional enrichment annotation analysis applying the KEGG, Reactome, and GO_BP databases was also carried out that identified several processes that were involved in the regulation of apoptosis, cell cycle, senescence, and the PI3K cascade (Supplementary Table S3).

### 3.4. The Application of miR-30d-5p Mimic Decreased Cell Proliferation in Both PEO1 and A2780 Ovarian Cells

**Figure 5:**
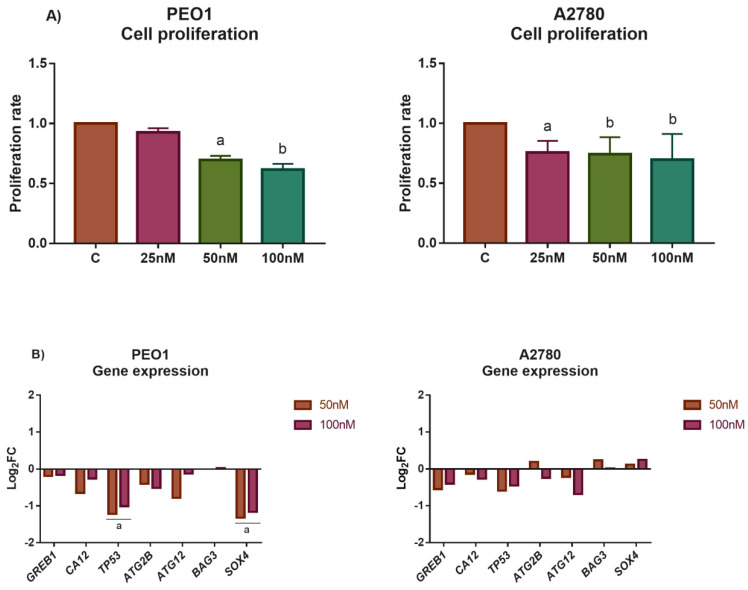
Study the effect of miR30d-5p mimic to the phenotype of PEO1 and A2780 cells. (**A**) Proliferation rate of cell cultures that were transfected with 25 nM, 50 nM, or 100 nM miR-30d-5p mimic. Cell proliferation was determined 24 h after transfection relative to the non-treated control (1). Data are presented as the mean ± S.D. a: *p* < 0.05; b: *p* < 0.01. (**B**) Log_2_FC values of selected genes in response to transfection by 50 nM and 100 nM miR-30d-5p mimic. Total RNA was isolated from the cells 24 h after transfection. Significance was determined between the ∆CT values of the treated and non-treated samples. a: *p* < 0.05.

MiR-30d-5p was chosen for further analysis on the grounds of the following results: (i) it showed high basal expression in PEO1 and responded well to high-dose E2 treatment where intensive cell death was observed, (ii) the functional annotation of miR-30d-5p targets resulted in several pathways that were involved in the regulation of cell cycle and cell death, and (iii) the application of miR-30d-5p mimic reduced cell proliferation and induced cell death in several cancer cell lines according to previous studies [28,33,34,35]. Based on these observations, we aimed to characterize the impact of miR-30d-5p in human ovarian cells by transfecting the PEO1 and A2780 cells with miR-30d-5p mimic. Successful transfection was confirmed by qPCR that showed a significant increase in miR-30d-5p level in the transfected cells (Log_2_FC values were 8.56 and 8.65 after transfection with 50 or 100 nM mimic compared to the non-transfected control in PEO1; and 6.69 and 8.52 in A2780). According to our results, the miR-30d-5p mimic significantly suppressed cell proliferation in both cell lines in a dose-dependent manner (Figure 5A), while cell proliferation was not affected significantly when the cells were transfected with the negative control in the same doses (Supplementary Figure S4). Gene expression studies revealed that the expression of *TP53* and *SOX4* was significantly decreased in the transfected PEO1 cells, which is in good agreement with the fact that these genes are considered to be targeted by miR-30d-5p [28,36]. The expression of the additional genes that were tested was not altered significantly (Figure 5B).

### 3.5. The Application of miR-30d-5p Mimic or AZD8835-Sensitized PEO1 Cells to Cell Death Induced by High-Dose E2 Treatment

**Figure 6:**
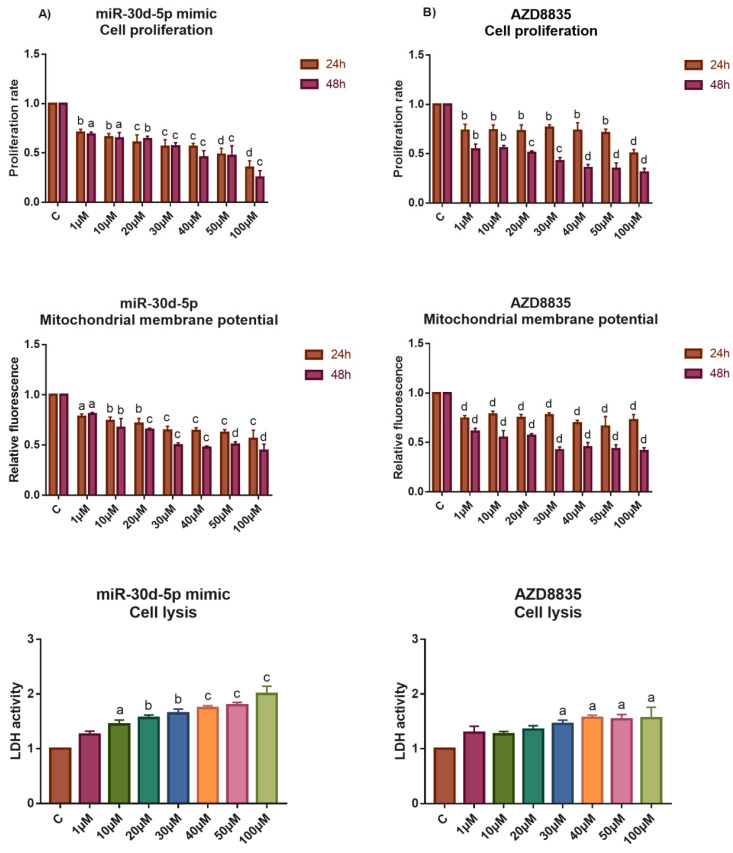
Study the effect of high-dose E2 exposure to the cell proliferation, apoptosis and cell lysis of the PEO1 cell cultures in the presence of miR-30d-5p mimic or AZD8835. Phenotypic characterization of high-dose E2 treatment was made in the PEO1 cell line in the presence of 50 nM miR-30d-5p mimic (**A**) or 100 nM AZD8835 (**B**). The rate of cell proliferation, mitochondrial membrane potential (24 h and 48 h after the treatment), and LDH activities (48 h after the treatment) were determined relative to the non-treated control (1). Data are presented as the mean ± S.D. a: *p* < 0.05; b: *p* < 0.01; c: *p* < 0.001; d: *p* < 0.0001.

**Figure 7:**
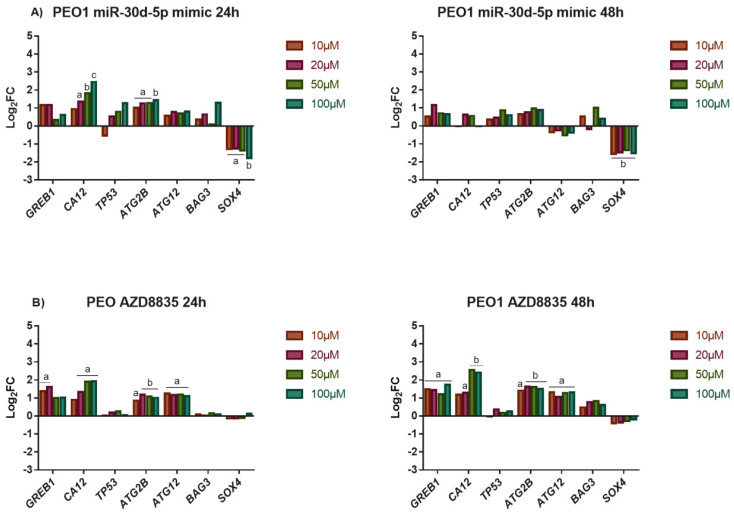
Study the effect of high-dose E2 exposure on gene expression in the presence of miR-30d-5p mimic or AZD8835. Log_2_FC values of selected genes were determined in response to E2 treatment in the PEO1 cell line in the presence of 50 nM miR-30d-5p mimic (**A**) or 100 nM AZD8835 (**B**). Total RNA was isolated from the cells at 24 h and 48 h after E2 treatment. Significance was determined between the ∆CT values of the treated and non-treated samples. a: *p* < 0.05; b: *p* < 0.01; c: *p* < 0.001.

The tolerance of ovarian cells that were transfected with miR-30d-5p mimic against high-dose E2 treatment was also tested. In these experiments, only the PEO1 cell line was used due to the fact that A2780 possessed high basal sensitivity to cell death that was induced by high-dose estrogens. As Figure 6A shows, transfecting the cells with 50 nM of the miR-30d-5p mimic sensitized the PEO1 cells to high-dose E2 treatment that was observable at 24 h and 48 h after the treatments as well. This result might be explained by the fact that miR-30d-5p interfered with the ERα-mediated estrogen response that was suggested by the significantly lower expression of *GREB1* and *CA12* genes compared to the E2-treated cells (Figure 3A and Figure 7A). Furthermore, *SOX4* showed marked repression in these cultures (Figure 7A). Although the expression of *TP53* and *ATG12* was not affected in these cells, *ATG2B* showed elevated expression even at 10 µM E2 (Figure 7A).

The miR-30d-5p mimic was reported to reduce cell proliferation by inhibiting the PI3K/AKT pathway through *SOX4* in pancreatic cancer cells [28]. Due to the observation that the PI3K/AKT pathway is important in the regulation of cell proliferation and considered to have a close interaction with the ERα-mediated estrogen response [37], we hypothesized that miR-30d-5p might have an effect on the ERα-mediated response through the PI3K/AKT pathway by targeting *SOX4*. In order to prove this hypothesis, we investigated the effect of high-dose E2 on PEO1 cells in the presence of AZD8835, which is a commercially available PI3K inhibitor [38]. This molecule was confirmed to inhibit PI3K-mediated signalling and cell proliferation in both breast and ovarian cancer cell lines in previous studies [38,39]. As expected, the application of AZD8835 reduced the tolerance of PEO1 cells against high-dose E2 (Figure 6B). The significantly lower induction of *GREB1* and *CA12* suggests that inhibiting the PI3K/AKT pathway interfered with ERα-mediated response (Figure 7B). It is also important to mention that the sustained induction of autophagy that was related *ATG2B* and *ATG12* genes was also observed in these cultures (Figure 7B). Note that the application of 100 nM AZD8835 solely did not affect the expression of miR-30s or the genes that were tested (Supplementary Table S2).

### 3.6. Reducing the Activity of the PI3K/AKT Pathway by miR-30d-5p Mimic or AZD8835 Sensitized PEO1 Cells to Tamoxifen

**Figure 8:**
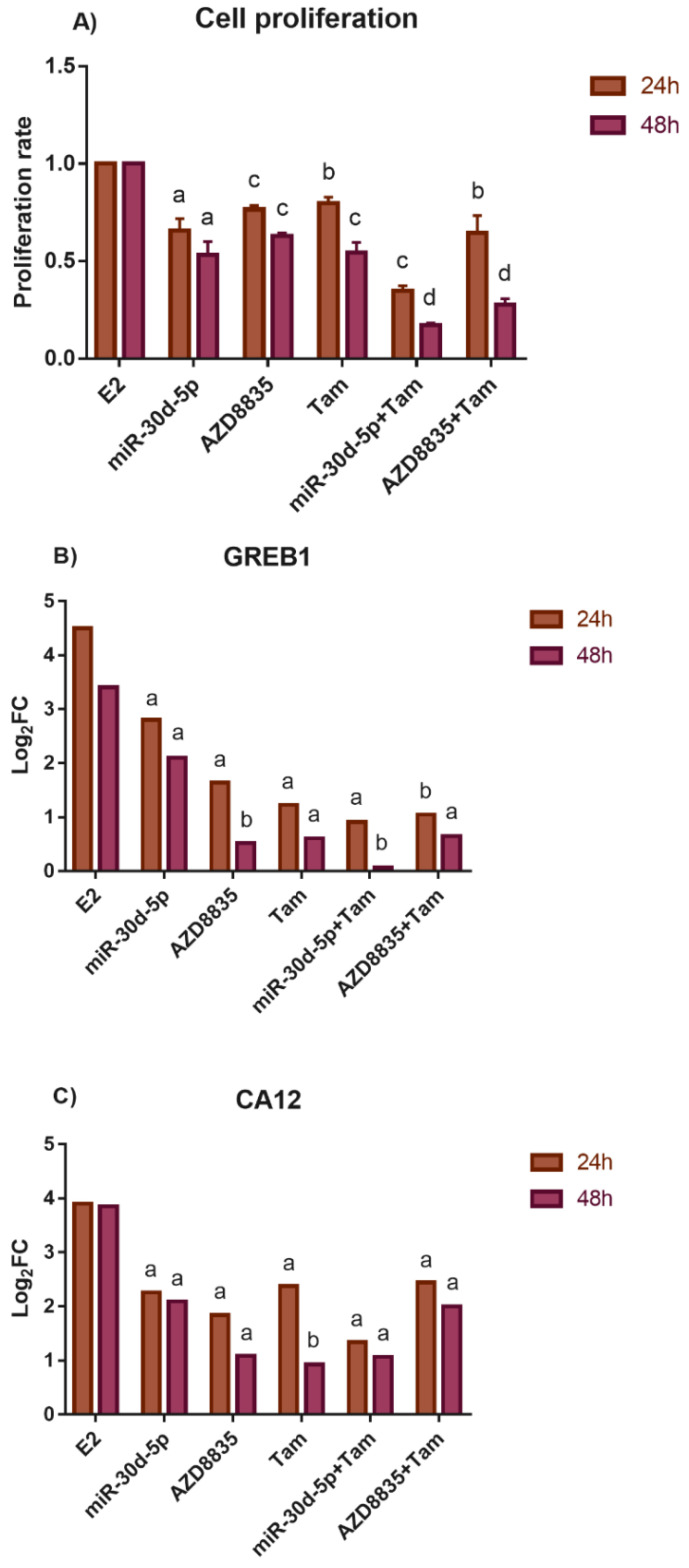
Study the effect of miR-30d-5p mimic and AZD8835 to the tamoxifen sensitivity of the PEO1 cell cultures. The proliferation rate (**A**) and the expression of *GREB1* (**B**) and *CA12* (**C**) genes were tested in E2 (10 nM)-treated PEO1 cultures that were supplemented with tamoxifen (Tam; 1 µM) and/or miR-30d-5p mimic (50 nM) and AZD8835 (100 nM). Cell proliferation and gene expression were determined 24 and 48 h after the treatments relative to the cells that were treated with E2 only. Cell proliferation data are presented as the mean ± S.D. Gene induction is presented as log_2_FC values. a: *p* < 0.05; b: *p* < 0.01; c: *p* < 0.001; d: *p* < 0.0001.

In the end of our work, we studied whether the application of the miR-30d-5p mimic decreased the tamoxifen sensitivity (that acts as an anti-estrogen) of PEO1 due to the following reasons: (i) a close interaction is suggested between the PI3K pathway and ER signaling in our study that was also supported previously in breast cancer [40], (ii) the concurrent inhibition of both PI3K and ER signaling proved to have synergistic effect and is considered to be a promising strategy in the therapy of breast cancer [40,41], and (iii) tamoxifen is considered to be a therapeutic option in ER+ ovarian cancer [42]. These experiments were carried out in the presence of 10 nM E2, which is considered to be a physiologically relevant E2 dose, and successfully induced proliferation as well as an ERα-mediated estrogen response previously [18]. According to our results, miR-30d-5p, AZD8835, as well as tamoxifen successfully inhibited the proliferative effect of 10 nM E2 that was in good agreement with the reduced induction of *GREB1* and *CA12* expression (Figure 8). Furthermore, the combination of tamoxifen with either miR-30d-5p or AZD8835 resulted in a more significant inhibition of cell proliferation (Figure 8A).

## 4. Discussion

Estrogens are Janus-faced molecules that are able to both trigger and inhibit cell proliferation in a dose-dependent manner. Cell proliferation is induced in nanomolar concentrations of E2 but at higher doses—in micromolar concentrations—E2 inhibits cell proliferation and induces apoptosis instead. ERs were shown to be important in the regulation of both processes [24,43]. ER-mediated induction of apoptosis is well-known in breast cancer cells that involve the extensive accumulation of unfolded proteins due to endoplasmic reticulum stress that leads to apoptosis through the intrinsic pathway, but the subsequent recruitment of the extrinsic pathway is also involved in the completion of the process [24]. Furthermore, an ER-independent apoptotic pathway was also identified involving phosphodiesterase 3A that results in the stabilization of SLFN12 protein and the blocking of translation [44]. However, little is known about the effect of high-dose estrogens in ovarian cells. We presented here that estrogens in micromolar concentrations were able to induce cell death in human ovarian cells, the process being mediated by the induction of apoptosis and autophagy. Apoptosis proved to be more relevant in the PEO1 cell line and autophagy was more significant in A2780, especially through the activity of *BAG3*. Both *TP53* and *BAG3* genes are considered to be under the regulation of ERα and ERβ [25,26,45,46]. We have to note that according to the known *TP53* mutation status of PEO1, we can make only careful conclusions about the *TP53*-mediated apoptosis of this cell line [47]. The most relevant observation in our study is that the presence of ERα-mediated response highly increases the tolerance of ovarian cells against high-dose E2. In the absence of the ERα-mediated response or when it was blocked by MPP in the PEO1 cell line, cell death was observed even at 1–10 µM which is in good agreement with the sensitivity that was observed in several other cell lines [44,48,49]. However, this dose was shown to be well-tolerable in ERα-expressing ovarian cells. This might be explained by the fact that E2 was able to induce an estrogen response even at 10 µM that might have contributed to the maintenance of cell proliferation. It is noteworthy that ~1–10 µM concentration of E2 is usually attained in a dominant follicle [50]. Thus, ovarian surface epithelial cells—that express ERα—are physiologically exposed to this concentration. It is also important to mention that ERα-mediated signaling was reported to be important in the suppression of autophagy in endometrial stromal cells and in neurons, and the knockdown of this receptor induced autophagy in MCF7 cells [51,52,53].

MiR-30 family members are considered to be tumor suppressors that are involved in the regulation of cell death and proved to be promising therapeutic molecules in several cancers [21]. According to their high basal expression, these molecules might have higher biological relevance in the ERα-expressing PEO1 cell line. A similar phenomenon was observed in breast cancer where the expression of miR-30s was elevated in ER+ cells compared to ER- samples [54,55]. However, these miRNAs did not respond to lower E2 doses where proliferation is induced, which rejects the assumption that these miRNAs are involved in the maintenance of proliferation. We suggest that miR-30a-5p, miR-30d-5p, and miR-30e-5p are involved in the regulation of cell death and senescence instead in ovarian cancer cells that was also suggested in other cancer types [21]. This assumption is supported by our results showing their marked induction in response to high-dose E2 in the PEO1 ovarian cells that was consistent with *TP53* upregulation. It is important to mention that miR-30 family members are considered to be in close synergy with *TP53* in the regulation of cell death. *TP53* is targeted by these miRNAs [36]. Recent studies have suggested that the transcription of miR-30 family members was upregulated by *TP53* and their coordinated induction contributed to the regulation of cell proliferation and invasion in cancer cells [56,57,58].

We also provide evidence that the transfection of miR-30d-5p mimic to ovarian cells reduced cell proliferation in both of ERα expressing and non-expressing cell lines. MiR-30d-5p reduced cell proliferation and induced apoptosis in pancreatic, ovarian granulosa, colon cancer cells, and in renal cell carcinoma as well regardless of ERα expression [28,33,34,35]. Ours is the first study that presents that transfection with miR-30d-5p suppresses proliferation in human ovarian cancer cells hinting that, in the future, it might be applied as a therapeutic agent in ovarian cancer. We also present that the application of this miRNA sensitized ovarian cells to cell death-inducing factors (e.g., to high-dose E2 treatment). The reduced response of *GREB1* and *CA12* to E2 exposure in the transfected PEO1 cells suggests that the miR-30d-5p mimic might have interfered with ERα signaling and suppressed the proliferative effect of E2. This might have been mediated by the *SOX4*/PI3K/AKT axis that was previously identified in pancreatic cells [28]. This is in line with the observation that the PI3K/AKT pathway is in close interaction with ERα signaling [37,40]. Furthermore, the PI3K/AKT pathway is highly involved in the development of multidrug resistance in cancer, including ovarian cancer, thus the inhibition of this pathway is considered to be a promising therapeutic strategy [59,60]. Combination therapies that direct both the PI3K and ER pathways proved to be applicable in the treatment of ER+ breast cancer [38,41,61,62,63]. According to our results such a therapy might be applicable in ovarian cancer as well due to the fact that blocking the PI3K pathway either by miR-30d-5p or by AZD8835, sensitized the cells to tamoxifen. It is also important to mention that the synergistic cytotoxicity of AZD8835 with cisplatin and carboplatin was observed in several human ovarian cell lines previously [39].

## 5. Conclusions

Chemotherapeutic resistance represents a great challenge in cancer treatment. Regarding ovarian cancer, the therapy is further complicated by intratumor heterogeneity. The development of new strategies as well as their combination with the existing therapeutic options represents promising directions in future cancer treatment. According to the fact that miR-30d-5p was able to inhibit proliferation in both of the applied cell lines, this miRNA might be a promising therapeutic molecule in tumors that are heterogenic for ERα expression. The fact that miR-30d-5p might interfere with ERα signaling further increases its clinical relevance because targeting this pathway is considered to be a promising therapeutic strategy in gynecological cancers (e.g., by the application of antiestrogens, aromatase inhibitors, or by the sustained activation of ERα) [7,64]. Furthermore, the application of high-dose estrogen as a treatment was also tested previously in tamoxifen-resistant breast cancer [24]. Our results suggest that miR-30d-5p alone or in combination with other drugs (e.g., with tamoxifen) might be a promising candidate for inhibiting cell proliferation and/or interfering estrogen signaling in ovarian cancer.

## Figures and Tables

**Table 1:** Degree and betweenness centrality values of miR-30d-5p and its targets in the miRNA-gene and protein–protein interaction network that were generated by the miRNet tool. The degree of a node presents the number of connections it has to neighboring nodes. Betweenness centrality values present the number of shortest paths that go through the node of interest in the network. The top 10 hits are presented.

Id	Degree	Betweenness
hsa-mir-30d-5p	368	68,210
*TP53*	29	446
*MYC*	19	77
*CAND1*	17	304
*EZH2*	12	91
*DCUN1D1*	12	29
*UBE2D3*	11	25
*NOTCH1*	9	15
*MCL1*	8	94
*MAPK8*	8	7

## Supplementary Materials

The following supporting information can be downloaded at: https://www.mdpi.com/article/10.3390/biomedicines10092060/s1. Figure S1: Study the effect of PHTPP (ERβ antagonist) to the A2780 cell line. (A) Proliferation rate of A2780 cells in the presence of PHTPP (10 nM–10 µM). (B) Proliferation rate of A2780 cells that were co-treated with 10 µM E2 and PHTPP 10 nM–10 µM. Cell proliferation was determined relative to the non-treated control (1). Data are presented as the mean ± S.D. a: *p* < 0.05; c: *p* < 0.001; d: *p* < 0.0001. Figure S2: Target analysis of miR-30s. Shared targets of miR-30a-3p, miR-30a-5p, miR-30d-5p, and miR-30e-5p were assessed by the miRNA Enrichment and Annotation (miEAA 2.0) webtool using the miRTarBase v8.0 database, Figure S3: Functional enrichment analysis of the molecular pathways where the targets of miR-30s are significantly enriched according to the miRPathDB tool using the miRTarBase 8.0 database, Figure S4: Proliferation rate of PEO1 and A2780 cells that were transfected with 25 nM, 50 nM, or 100 nM microRNA Mimic Negative Control. Cell proliferation was determined relative to the non-treated control (1). Data are presented as the mean ± S.D. Table S1: Primer sequences for the qPCR analysis of mRNAs. Table S2: FC values of miR-30s and tested genes in the PEO1 cells that were treated with 10 nM MPP or 100 nM AZD8835. MiRNA and mRNA expression was determined relative to the non-treated control (1). Table S3: Functional enrichment annotation analysis of the gene–gene and protein–protein interactions of miR-30d-5p targets applying the KEGG, Reactome and GO_BP databases.
