# Supplementary material for: Suppressing the PI3K/AKT Pathway by miR-30d-5p Mimic Sensitizes Ovarian Cancer Cells to Cell Death Induced by High-Dose Estrogen
Source: Biomedicines. 2022 Aug 24;10(9):2060. doi: 10.3390/biomedicines10092060 (PMC9495868; doi:10.3390/biomedicines10092060)
Supplement: Supplementary file 1 [file biomedicines-10-02060-s001.zip › biomedicines-1785897-supplementary-.pdf]

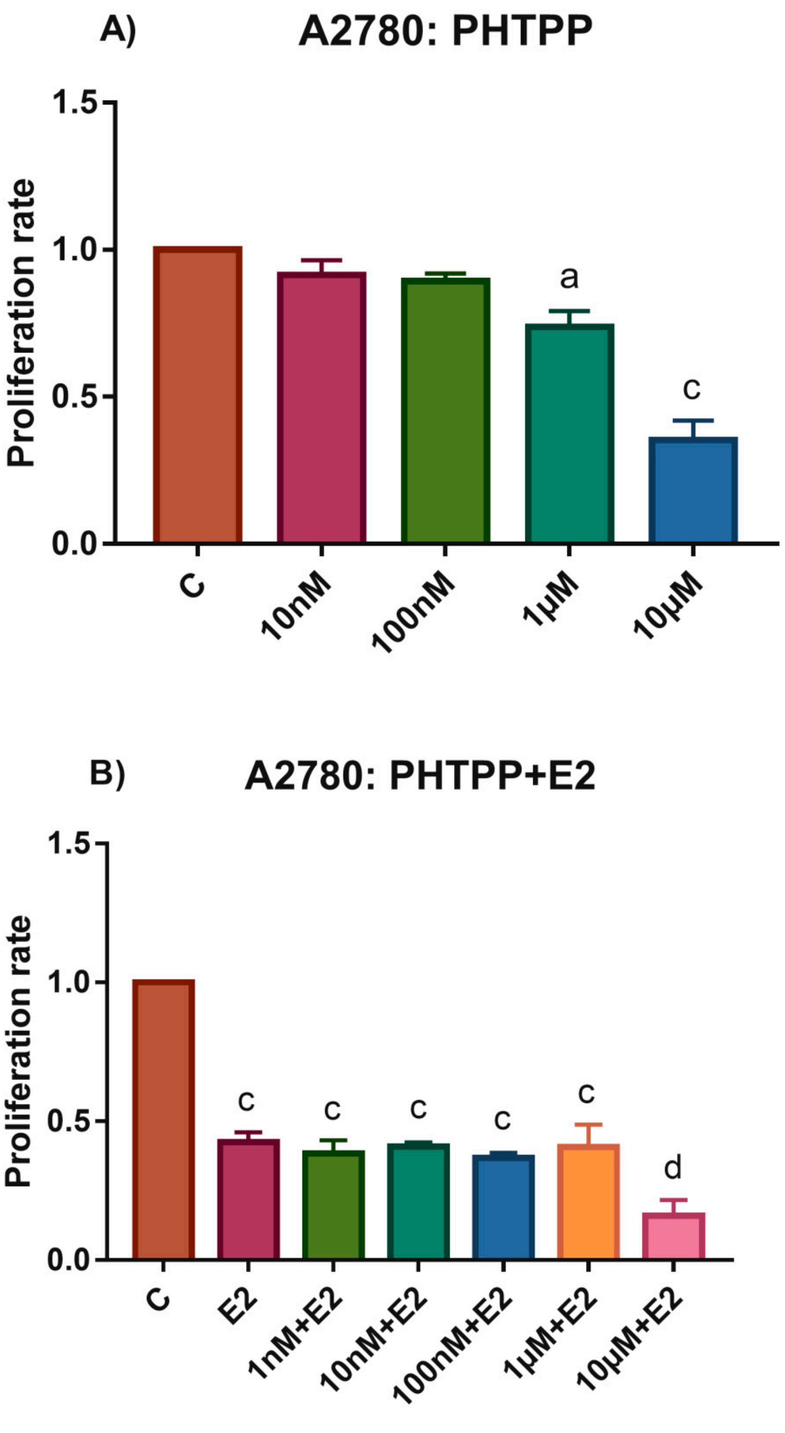

Supplementary Figure S1 Study the effect of PHTPP (ERβ antagonist) to the A2780 cell line. A) Proliferation rate of A2780 cells in the presence of PHTPP (10nM-10µM). B) Proliferation rate of A2780 cells co-treated with 10µM E2 and PHTPP 10nM-10µM. Cell proliferation was determined relative to the non-treated control (1). Data are presented as mean ± S.D. a: $p<0.05$; c: $p<0.001$; d: $p<0.0001$

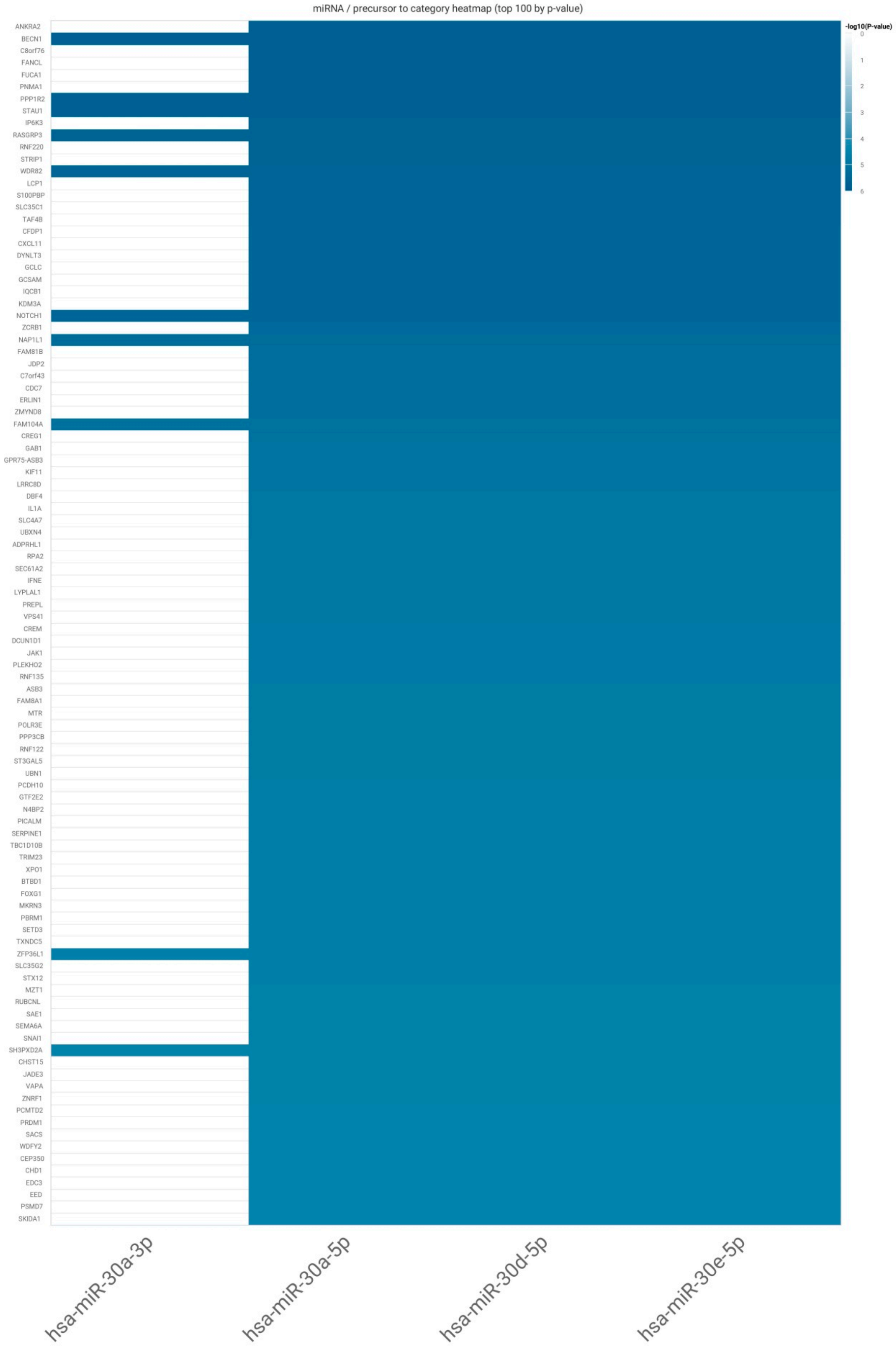

Supplementary Figure S2 Target analysis of miR-30s. Shared targets of miR-30a-3p, miR-30a-5p, miR-30d-5p and miR-30e-5p that was assessed by the miRNA Enrichment and Annotation (miEAA 2.0) webtool using the miRTarBase v8.0 database.

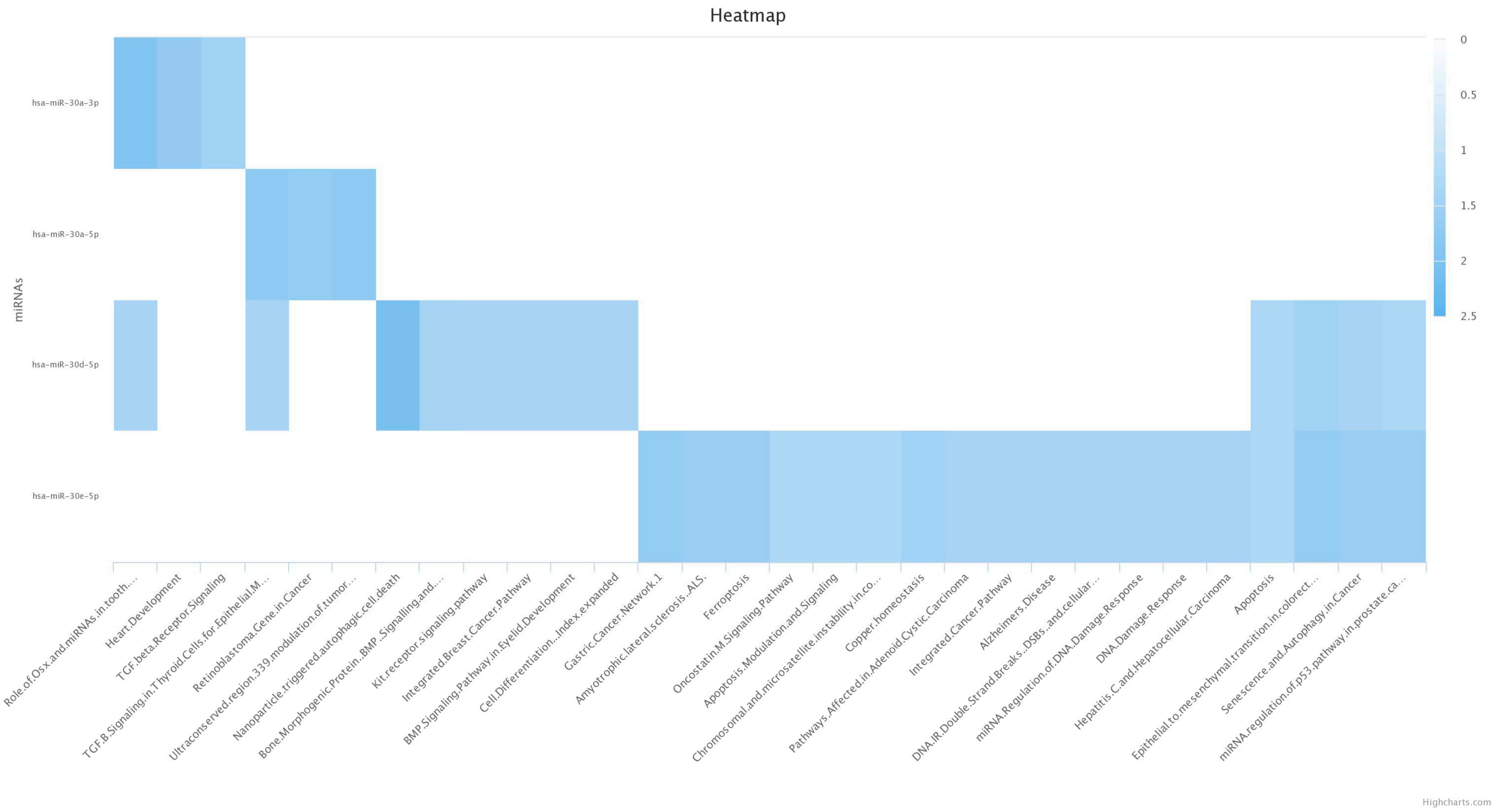

Supplemntary Figure S3 Functional enrichment analysis of the molecular pathways where the targets of miR-30s are significantly enriched according to the miRPathDB tool using the miRTarBase 8.0 database.

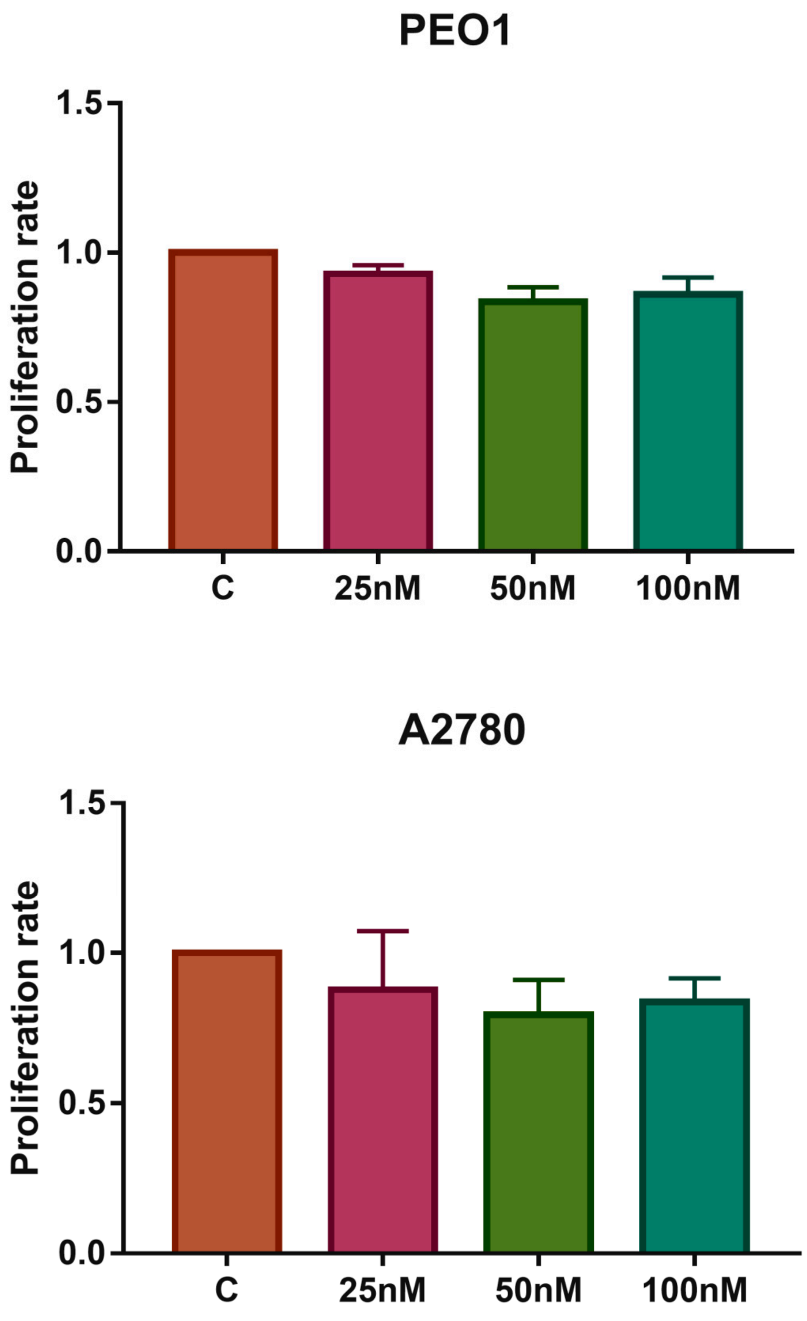

Supplementary Figure S4 Proliferation rate of PEO1 and A2780 cells transfected with 25 nM, 50 nM or 100 nM microRNA Mimic Negative Control. Cell proliferation was determined relative to the non-treated control (1). Data are presented as mean ± S.D.

Supplementary Table S1. Primer sequences for the qPCR analysis of mRNAs.

	**Forward**	**Reverse**
GREB1	TCTTGCACAATTCCATCGAG	GTCCACTCGGCTACCACCT
CA12	GTTTCTCCTGACCAACAATGG	CGTGGCACTGTAGCGAGAC
TP53	ACACGCTTCCCTGGATTG	GCTCGACGCTAGGATCTGAC
ATG2B	GGAGTGTCTCTCTTCTGGGATG	AGGTGAGAGCTTTGGCTCAG
ATG12	TCTTCCGCTGCAGTTTCC	GGAGCAAAGGACTGATTCACA
BAG3	CCCCGTTCAGGTCATCTGTC	AGGTGCAGTTTCTCGATGGG
SOX4	GACCTGAACCCCAGCTCAAA	AGCCGGGCTCGAAGTTAAAA
ESR2	TGTTAATGATGGGGCTGATGT	CTTCTACGCATTTCCCCTCA
GAPDH	CACCCACTCCTCCACCTTT	GCCAAATTCGTTGTCATACCA

Supplemetray Table S2 FC values of miR-30s and tested genes in the PEO1 cells treated with 10 nM MPP or 100 nM AZD8835. MiRNA and mRNA expression was determined relative to the non-treated control (1).

	**10 nM MPP**	**100 nM AZD8835**
miR-30a-3p	0.71	0.72
miR-30a-5p	0.87	1.22
miR-30d-5p	1.09	0.98
miR-30e-5p	0.73	1.11
GREB1	0.81	0.82
CA12	1.2	0.84
TP53	0.9	0.74
ATG2B	1.19	0.95
ATG12	1.12	1.21
BAG3	1.02	0.78
SOX4	0.85	0.79
